# Prospective Analysis of Short- and Mid-term Knowledge Retention after a Brief Ultrasound Course for Undergraduate Medical Students

**DOI:** 10.6061/clinics/2019/e1087

**Published:** 2019-09-10

**Authors:** Carlos Augusto M Menegozzo, Priscila Gadelho Cazolari, Fernando da Costa Ferreira Novo, Ramiro Colleoni, Edivaldo Massazo Utiyama

**Affiliations:** I Disciplina de Cirurgia Geral e Trauma, Hospital das Clinicas HCFMUSP, Faculdade de Medicina, Universidade de Sao Paulo, Sao Paulo, SP, BR; II Universidade Federal de Sao Paulo, Sao Paulo, SP, BR; III Departamento de Cirurgia, Universidade Federal de Sao Paulo, Sao Paulo, SP, BR

**Keywords:** Medical Education, Medical Student, Ultrasonography, Point-of-Care Technology

## Abstract

**OBJECTIVES:**

The benefits of implementing point-of-care ultrasound (POCUS) in the emergency department are well established. Ideally, physicians should be taught POCUS during medical school. Several different courses have been designed for that purpose and have yielded good results. However, medical students need specifically designed courses that address the main objectives of knowledge acquisition and retention. Despite that, there is limited evidence to support knowledge retention, especially in the mid-term. The purpose of this study is to evaluate short- and mid-term knowledge retention after a student-aimed ultrasound course.

**METHODS:**

Medical students participating in a medical student trauma symposium (SIMPALT) in 2017 were included. Their profiles and baseline ultrasound knowledge were assessed by a precourse questionnaire (PRT). The same questionnaire was used one week (1POT) and three months (3POT) after the course.

**RESULTS:**

Most of the participants were 1^st^- to 4^th^- year medical students. None had prior ultrasound knowledge. They reported costs as the major barrier (65%) to enrollment in an ultrasound course. A comparison between the PRT and 1POT results showed a statistically significant difference (*p*<0.02), while no difference was found between 1POT and 3POT (*p*>0.09).

**CONCLUSION:**

Our findings support the use of a tailored ultrasound course for medical students. Knowledge acquisition and mid-term retention may be achieved by this specific population.

## INTRODUCTION

Point-of-care ultrasound (POCUS) is becoming an integral part of the assessment of critical care patients. It is considered “the new stethoscope” by some authors 1,2 and is a useful tool for the evaluation of abdominal pain, intracranial hypertension, pleuropulmonary diseases, shock, and airway control 3,4.

As with any technology with widely expanding applications, it is imperative that physicians be well trained in POCUS. Moreover, there is an ongoing debate regarding whether training in POCUS should be offered to medical students. FAST (Focused Assessment with Sonography for Trauma) is one of the simplest applications of POCUS. Free fluid identification is straightforward due to the contrast with the adjacent structures and does not depend on artifact interpretation 5. Hence, FAST is a good example to present to medical students as the first step in ultrasound training.

Various POCUS courses are reported in the literature 6-11, some of which are aimed at medical students 12-17. However, their use is limited by factors such as duration and financial cost. Most courses assess knowledge acquisition using a questionnaire administered shortly after course completion. However, there is little evidence to support knowledge retention in the mid-term. This gap may undermine the interpretation of course efficacy and contribute to a lower enrollment rate.

An ideal tailored course for medical students should be short and inexpensive while resulting in satisfactory knowledge retention. Hence, the present study aims to evaluate the effects of a brief, student-tailored ultrasound course by focusing on short- and mid-term knowledge retention.

## METHODS

This is a nonrandomized prospective study that included participants who enrolled in the skills stations at a medical student trauma symposium (SIMPALT) in 2017. The ultrasound course was one of the four skills stations of the symposium. The students were separated into four groups that rotated through the stations every 50 minutes.

The ultrasound course encompassed basic theoretical explanations of ultrasound physics, transducer choice, FAST systematization, E-FAST windows, and image interpretation. The theoretical session was followed by practical training in performing a FAST exam of a healthy volunteer. The course format was elaborated by one of the authors (CAMM). Every student received two questionnaires before the course. The first was intended to gather participants' profile information and to determine what they considered barriers to enrolling in ultrasound courses. The second questionnaire (PRT) was composed of 8 multiple-choice theoretical questions, each of which had a unique correct answer, with the aim of assessing the students' baseline knowledge. We excluded participants who did not answer both questionnaires from further evaluation.

Every student received the same questions one week (1POT) and three months (3POT) postcourse. Responses were compared between PRT and 1POT to evaluate knowledge acquisition and between 1POT and 3POT to evaluate knowledge retention in the mid-term. Students were also asked to grade the overall quality of the activity on a scale of 1 to 10.

We performed chi-square and Fisher's exact tests using STATA software (STATACorp. 2007. Stata Statistical Software: Release 10.0. College Station, Texas: Stata Corporation) to compare the responses. The confidence interval was 95%, and *p*-values <0.05 were considered statistically significant.

## RESULTS

This study was approved by the Institutional Ethics Committee and is reported according to the STROBE guidelines. Thirty-seven students answered the first two questionnaires and were eligible for inclusion. Table 1 presents the profile information of the included students and their responses regarding the main barriers to enrollment in an ultrasound course. The response rates for 1POT and 3POT were 49% and 32%, respectively. None of the students had participated in previous ultrasound courses. The mean course satisfaction score was 9.03 out of 10.

A comparison of the PRT and 1POT responses showed overall knowledge acquisition, which was statistically significant for 6 of the 8 questions (Table 2). A comparison between 1POT and 3POT revealed no significant difference despite a mild decrease in correct answers (Table 3). This finding was correlated with knowledge retention in the mid-term (3 months).

## DISCUSSION

The results of this study support the effectiveness and feasibility of a brief student-tailored ultrasound course. Comparisons of the answers to the questionnaires show acquisition and mid-term retention of knowledge.

The current study confirmed previous observations of the perceived barriers to ultrasound course enrollment. Financial investments, routine curricular activities, course location and time spent on the course were the main barriers. This brief course was also low-cost (less than US$ 30), included in symposium's main scientific program, and performed at the same site. Hence, the main barriers were overcome. Some studies evaluate factors associated with limited incorporation of POCUS. One of the most important factors is the lack of training 18,19. However, evidence regarding a specific analysis of barriers to course enrollment is lacking. This unexplored subject may undermine the development of newer educational platforms 20. Our study provides potentially useful results to enhance students' participation in courses.

The optimal time to introduce POCUS concepts during medical education is still a matter of debate. The American Academy of Emergency Medicine advocates offering POCUS training to medical students 2,21,22. In fact, the incorporation of ultrasonography is well accepted among students who recognize various applications of that technology 15,23. Brunner et al. 24 in 1995, debated the introduction of ultrasound concepts to medical students by using echocardiography as an adjunct to the cardiac physiology course. The author demonstrated that echocardiography received the best rating among several topics of the course. This success may be related to a unique ability of ultrasound: increased integration of other subjects, such as anatomy, physiology, radiology, and surgery. FAST is an excellent example of such integration and is easily reproducible.

Studies have shown that medical students are capable of using ultrasound. In a study by Gogalniceanu et al., UK medical students demonstrated 88% accuracy in identifying free peritoneal fluid after a 5-hour POCUS course 18. Additionally, participants reported overall improvement of their knowledge regarding radiological anatomy and interest in further ultrasound training. They stressed the need to have this training widely available during medical school. Indeed, there are several benefits associated with such curriculum modifications. Barriers such as costs and the search for an adequate course would likely disappear. Additionally, ultrasound education for medical students would be homogeneous and standardized.

Several studies have analyzed knowledge acquisition by medical students after an ultrasound course. However, as noted in Table 4, none evaluated its retention in the mid- or long-term 20. Our results highlight the mid-term efficacy of a short and straightforward ultrasound course. Another interesting finding of our study is that the majority of the participants were enrolled in the preclinical stages of medical education. This result supports the ability of students in the early phases of medical school to acquire and retain knowledge.

### Limitations

The evaluation of knowledge based on theoretical questions, and no practical evaluation was performed. Hence, we could not assess mid-term knowledge retention in terms of actual performance of the exam. The participants' response rate decreased during the study, and only 32% completed the 3POT questionnaire. This means that a potential significant difference may not have been detected and that there is a risk of selection bias. Moreover, we could not compare the results to a control group because every student participated in the course. Last, we did not determine whether the students had gathered information from other sources during the 3-month interval between the two questionnaires (1POT and 3POT), although this was unlikely.

## CONCLUSION

This study makes two main contributions. First, a brief student-tailored ultrasound course results in knowledge acquisition and mid-term retention. Second, we demonstrated that costs, release from routine activities, location, and duration may undermine course enrollment. Factors impacting the dissemination and routine application of POCUS should be systematically assessed. The adoption of structured POCUS courses for medical students depends on a better understanding of the results of such training. We should make efforts to establish effective educational strategies to avoid potential barriers to course enrollment. Further prospective studies evaluating the impact of mid-term knowledge retention on the development of practical skills must be designed.

## AUTHOR CONTRIBUTIONS

Menegozzo CAM was responsible for the study design, data collection, literature review and manuscript writing. Cazolari PG was responsible for the data collection and manuscript writing. Novo FCF was responsible for the study design. Colleoni R was responsible for the critical final review of the manuscript. Utiyama EM was responsible for the critical final review of the manuscript.

## Figures and Tables

**Table 1:** Profile of the participants according to their responses to one of the precourse questionnaires (n=37).

Age	
<20 years	12 (32%)
21-25 years	22 (60%)
>25 years	3 (8%)
Gender	
Male	13 (35%)
Female	24 (65%)
Year of medical education	
1^st^ and 2^nd^ years	18 (49%)
3^rd^ and 4^th^ years	15 (40%)
5^th^ and 6^th^ years	4 (11%)
Baseline familiarity with ultrasound equipment (more than one answer per student permitted)	
None	30 (79%)
Knows how to change transducers	3 (8%)
Knows how to choose the appropriate transducer	Zero
Knows basic features	2 (5%)
Knows advanced features	Zero
Barriers to enrollment in an ultrasound course (more than one answer per student permitted)	
Financial investment	24 (65%)
Release from routine activities	14 (38%)
Course location	14 (38%)
Course duration	9 (24%)

**Table 2:** Comparison of correct responses at PRT and 1POT.

Topic	PRT (n=37)	1POT (n=18)	*p*-value
Q1. US machine functionalities	26 (70%)	15 (83%)	0.346
Q2. Transducer selection	28 (78%)	15 (83%)	0.731
Q3. FAST acoustic windows	27 (73%)	18 (100%)	0.021
Q4. EFAST acoustic windows	10 (27%)	13 (72%)	0.001*
Q5. Comparison: X-ray *vs*. US	6 (17%)	10 (55%)	0.004
Q6. Free fluid identification	8 (22%)	12 (66%)	0.001*
Q7. Hepatorenal evaluation in FAST	8 (22%)	13 (72%)	<0.001*
Q8. Pericardial evaluation in FAST	2 (6%)	12 (66%)	<0.001

*p-*value using Fisher's exact test.

* *p-*value using the chi-square test.

**Table 3:** Comparison of correct responses at 1POT and 3POT.

Topic	1POT (n=18)	3POT (n=12)	*p*-value
Q1. US machine functionalities	15 (83%)	10 (83%)	0.999
Q2. Transducer selection	15 (83%)	10 (83%)	0.999
Q3. FAST acoustic windows	18 (100%)	10 (83%)	0.152
Q4. EFAST acoustic windows	13 (72%)	8 (66%)	0.999
Q5. Comparison: X-ray *vs*. US	10 (55%)	7 (58%)	0.999
Q6. Free fluid identification	12 (66%)	5 (41%)	0.119*
Q7. Hepatorenal evaluation in FAST	13 (72%)	5 (41%)	0.094*
Q8. Pericardial evaluation in FAST	12 (66%)	6 (50%)	0.361*

*p*-value using Fisher's exact test.

* *p-*value using the chi-square test.

**Table 4:** Studies analyzing the impact of an ultrasound course on the knowledge of medical students.

	Number of participants	Subject	Course duration	Evaluation of knowledge acquisition	Evaluation of knowledge retention
Arger 2005 17	33	Kidney and aorta	4 weeks	Yes	No
Kondrashov 2015 14	248	Mixed	Not specified	Yes	No
Wong 2011 13	13	Aorta	21 days	Yes	No
Bell 2015 15	20	Heart	Not specified	Yes	No
Florescu 2015 16	64	Mixed	6 days	Yes	No
Gogalniceanu 2010 18	26	FAST	Not specified	Yes	No
Menegozzo 2019 23	37	FAST	50 min	Yes	Yes
